# Increased GS‐II lectin binding and SATB2 downregulation are biological features for sessile serrated lesions and microvesicular hyperplastic polyps

**DOI:** 10.1111/pin.13321

**Published:** 2023-04-10

**Authors:** Hisanori Matoba, Mai Iwaya, Yoshiko Sato, Noriyasu Kobayashi, Haruka Takemura, Yusuke Kouno, Ayumi Karasawa, Jun Nakayama

**Affiliations:** ^1^ Department of Molecular Pathology Shinshu University School of Medicine Matsumoto Japan; ^2^ Department of Laboratory Medicine and Pathology Shinshu University Hospital Matsumoto Japan; ^3^ Department of Laboratory Medicine JA North Alps Medical Center Azumi Hospital Oaza‐ikeda Kitaazumi‐gun Japan; ^4^ Department of Pathology Ina Central Hospital Koshiroukubo Ina Japan

**Keywords:** GS‐II, microvesicular hyperplastic polyps, SATB2, sessile serrated lesions, βGlcNAc

## Abstract

Sessile serrated lesions (SSLs) and microvesicular hyperplastic polyps (MVHPs) are colorectal lesions displaying gastric differentiation. *Griffonia simplicifolia*‐II (GS‐II) is a lectin specific to terminal α/βGlcNAc residues. Here, we assessed GS‐II binding and performed immunostaining for HIK1083 (specific to terminal αGlcNAc residues), MUC5AC, MUC6, and special AT‐rich sequence binding protein 2 (SATB2) in SSLs, MVHPs, and tubular adenomas (TAs). We observed MUC5AC positivity in 28 of 30 SSLs, but in only three of 23 TAs. Moreover, 24 of 30 SSLs were MUC6‐positive, while none of the 23 TAs were MUC6‐positive. None of the 30 SSLs or 23 TAs showed HIK1083 positivity. All 30 SSLs and 26 MVHPs were GS‐II‐positive, while only seven of 23 were in TAs. GS‐II staining was mainly distributed in the Golgi region, but SSLs and MVHPs showed goblet cell distribution, in 20 of 30 and 19 of 26 cases, respectively. All SSLs, MVHPs, and TAs were SATB2‐positive, but 21 of 30 SSLs and 12 of 26 MVHPs showed decreased staining intensity relative to adjacent mucosa, a decrease seen in only two of 23 in TAs. These results indicate overall that increased terminal βGlcNAc and decreased SATB2 expression are characteristics of SSLs and MVHPs.

**Abbreviations**
CIMP‐H: CpG island methylator phenotype high
GS‐II: *Griffonia simplicifolia*‐II
H_2_O_2_
: hydrogen peroxide
HRP: horseradish peroxidase
IBD: inflammatory bowel disease
IHC: immunohistochemistry
MSI‐H: microsatellite instability high
MVHP: microvesicular hyperplastic polyp
SATB2: special AT‐rich sequence binding protein 2
SSL: Sessile serrated lesion
TA: Tubular adenoma

## INTRODUCTION

Sessile serrated lesions (SSLs) and microvesicular hyperplastic polyps (MVHPs) are precursor lesions of serrated pathway‐associated colorectal carcinomas. MVHPs reportedly occur with *BRAF* mutations, gradually progress to SSLs, and finally become colorectal carcinomas with microsatellite instability high (MSI‐H) and CpG island methylator phenotype high (CIMP‐H).^1^, ^2^, ^3^, ^4^, ^5^, ^6^ Histologically, SSLs and MVHPs are both characterized with prominent serrations of surface to central portion of crypts, and distinguished in that SSLs only have dilated, distorted, branching, and serrated crypt bases. SSLs, MVHPs, and serrated pathway‐associated colorectal carcinomas reportedly show mucin phenotypes of gastric differentiation. These lesions express MUC5AC (gastric surface mucous cell‐type mucin) and MUC6 (gastric gland mucous cell‐type mucin) as well as MUC2 (intestinal goblet cell‐type mucin) and show bidirectional gastric differentiation.^7^, ^8^, ^9^, ^10^, ^11^ Analyses of these lesions report minimal terminal αGlcNAc glycosylation (a sugar residue specific to gastric gland mucous cell‐type mucin) based on infrequent immunohistochemical positivity to the monoclonal antibody HIK1083. However, glycosylation of these mucin core proteins is unclear.

*Griffonia simplicifolia*‐II (GS‐II) is a lectin derived from *Bandeiraea simplicifolia* that binds specifically to terminal α/βGlcNAc residue.^12^ Previously, we reported that GS‐II binds to colorectal cancer cells with a binding frequency greater than that seen in high‐grade colorectal tubular adenoma (TA) cells.^13^, ^14^ However, there are as yet no reports of GS‐II binding in SSLs and MVHPs.

We also focused on the expression of special AT‐rich sequence binding protein 2 (SATB2), a nuclear matrix‐associated transcription factor and epigenetic regulator initially identified as functioning in osteoblast differentiation and craniofacial patterning in humans.^15^, ^16^ SATB2 is normally expressed in the large intestinal epithelium and serves as a diagnostic marker of colorectal adenocarcinoma.^17^, ^18^, ^19^ Recently, we and others observed reduced SATB2 expression in sessile serrated pathway‐associated colorectal carcinomas, inflammatory bowel disease (IBD) associated colorectal carcinomas and dysplasias,^20^, ^21^, ^22^ although SATB2 expression in SSLs, MVHPs, and TAs remained unclear.

Therefore, the aim of this study is to investigate GS‐II binding and SATB2 expression in SSLs and MVHPs, to identify novel biological features for these lesions, and to understand their potential function in serrated pathway tumorigenesis.

## MATERIALS AND METHODS

### Case selection

Thirty SSL cases, 26 MVHP cases, and 23 TA cases were identified in the pathology archive of JA North Alps Medical Center Azumi Hospital, Ina Central Hospital, and Shinshu University Hospital from 2017 to 2020. Histological diagnosis of SSLs, MVHPs, and TAs was made following the WHO Classification of Tumors, 5th edition, Volume 1, Digestive System Tumors.^23^ SSLs with dysplasia were excluded, and only SSLs without dysplasia were analyzed here. Normal colonic mucosa adjacent to the lesion was examined as needed.

Use of retrospective tissue samples was approved by the Ethics Committees of North Alps Medical Center Azumi Hospital (no. 476), Ina Central Hospital (no. 21‐4), and Shinshu University Hospital (no. 5148).

### Immunohistochemistry (IHC) and GS‐II‐horseradish peroxidase (HRP) staining

Histological and immunohistochemical studies were performed using 3 μm thick sections from formalin‐fixed and paraffin‐embedded tissue blocks. Sections were stained with hematoxylin and eosin. Reagents and procedures used for IHC and GS‐II‐HRP staining are shown in Supporting Information: supplementary Table 1. In IHC, tissue sections were deparaffinized and rehydrated through a series of xylene and ethanol. Endogenous peroxidase activity was blocked using hydrogen peroxide (H_2_O_2_) in methanol. Antigen retrieval was carried out by microwaving, as indicated in Supporting Information: supplementary Table 1, and slides were incubated for 1 h with primary antibodies. Secondary antibodies were from the EnVision+ System (anti‐mouse or anti‐rabbit) (Dako) and were incubated with slides for either 30 min or 1 h, depending on the primary antibody. Peroxidase activity was visualized using a diaminobenzidine‐H_2_O_2_ solution. GS‐II‐HRP staining was generally performed in the same way as IHC, although slides were incubated for 30 min with a 0.1% amylase solution to block GS‐II reactivity to glycogen after endogenous peroxidase activity blocking and HRP‐conjugated streptavidin (Dako) was used as a secondary antibody. Negative controls were: for GS‐II‐HRP staining, lectin solutions containing 0.1 mol/L GlcNAc, and for IHC, staining solutions lacking primary antibodies. No specific staining was seen in either negative control.

### GS‐II‐HRP staining with PNGase F digestion

Tissue sections were deparaffinized and rehydrated and incubated with 0.1% amylase solution for 30 min to block GS‐II reactivity to glycogen. After washing with water, sections were incubated with 5% PNGase F (New England Biolabs) containing 10× Glycoprotein Denaturing Buffer, 10× GlycoBuffer 2, and 10% NP‐40 at 37°C overnight. Endogenous peroxidase activity was blocked using H_2_O_2_ in methanol, and tissue sections were subjected to GS‐II‐HRP staining as described above. Two colonic adenocarcinomas and one pyloric gland mucosa sample surrounding gastric adenocarcinoma were used as positive and negative controls of the digestion, respectively.

### Evaluation of IHC and GS‐II‐HRP staining

The extent of staining for MUC5AC, MUC6, HIK1083, SATB2, and GS‐II was scored semiquantitatively (0 = no staining; 1 ≤ 5%; 2 = 5%–25%; 3 = 26%–50%; 4 = 51%–75%; 5 = 76%–100%), and maximum intensity was graded as negative, weak (weakly stained but judged negative in routine diagnosis), moderate (stained with normal intensity), or strong (apparently strongly stained), as previously described.^21^ For binary analyses, cases with 5% or more tumor cells showing moderate or strong intensity were considered positive. We judged that βGlcNAc was present if cases were HIK1083‐negative and GS‐II‐positive. Regional distributions of these markers to the upper crypt portion, crypt base, or entire crypt length were also evaluated. For SATB2, we also evaluated immunostaining intensities at lesion sites relative to adjacent colonic mucosa: for example, even if staining intensities at a lesion site and adjacent colonic mucosa were both judged moderate according to the above criteria, if intensities differed, we scored that as a decrease. For GS‐II‐HRP staining, distribution of GS‐II to goblet cells was also evaluated in GS‐II‐positive SSLs, MVHPs, and TAs. Two authors (HM, MI) reviewed immunohistochemical stains and reached a consensus score for each case.

### Statistical analysis

Fisher exact tests were used to compare the binary results of IHC and GS‐II‐HRP staining in SSLs, MVHPs, and TAs. The Holm method was used to correct for multiple comparisons. Differences were considered significant at *p* < 0.05. All statistical analyses were performed using EZR 1.37^24^ (Saitama Medical Center, Jichi Medical University), which is a graphical user interface for R (The R Foundation for Statistical Computing, Vienna, Austria, version 3.4.1).

## RESULTS

### MUC5AC, MUC6, and HIK1083 immunostaining in SSLs and TAs

**Figure 1:**
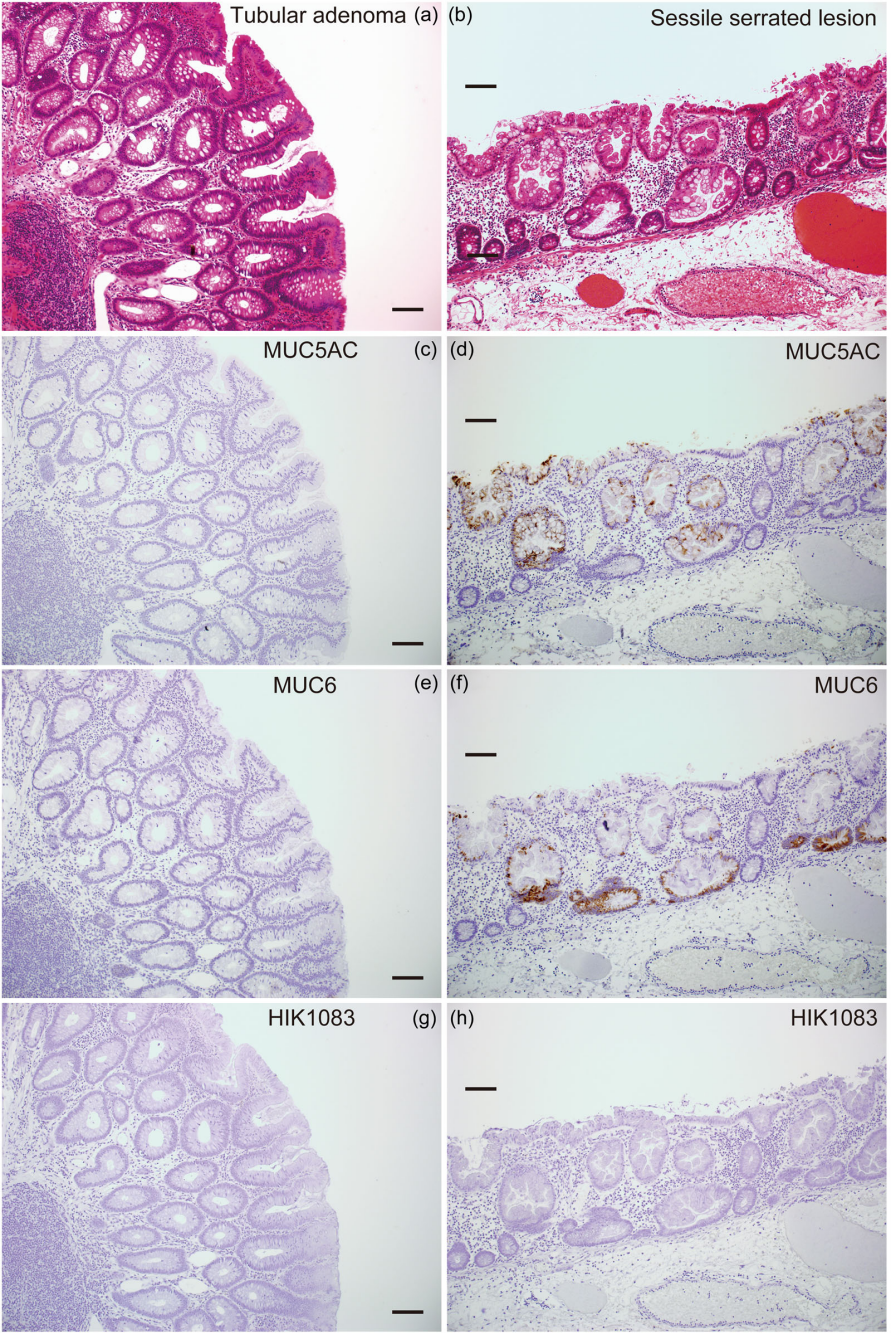
Gastric‐type mucin expression in tubular adenomas (TAs) and sessile serrated lesions (SSLs). (a, b) Hematoxylin and eosin staining in TAs (a) and SSLs (b). (c, d) Immunostaining for MUC5AC in TAs (c) and SSLs (d). (e, f) Immunostaining for MUC6 in TAs (e) and SSLs (f). (g, h) Immunostaining for HIK1083 in TAs (g) and SSLs (h). Scale bars, 100 μm (a–h).

We began by evaluating gastric differentiation in SSLs and TAs based on MUC5AC, MUC6, and HIK1083 immunostaining. MUC5AC was positive in 28 (93%) of 30 SSLs but in only three (13%) of 23 TAs (*p* < 0.001) (Table 1 and Figure 1c,d). MUC5AC‐positive cells were typically observed throughout the entire length of the crypt in SSLs. However, in one (4%) of 28 MUC5AC‐positive SSLs, we observed distribution mainly to the upper crypt. MUC6 was positive in 24 (80%) of 30 SSLs but in none (0%) of the 23 TA cases (*p* < 0.001) (Table 1 and Figure 1e,f). MUC6‐positive cells were observed in crypt bases in all MUC6‐positive SSL cases. Focal and weak HIK1083 immunostaining was observed in one SSL case and one TA case, but none of the 30 SSL and 23 TA cases showed significant positivity based on study criteria (Table 1 and Figure 1g,h). Overall, MUC5AC and MUC6 expression were upregulated primarily in SSLs, while terminal αGlcNAc glycosylation (detected by HIK1083 immunostaining) was not observed. These results are generally in agreement with previous studies.^7^, ^8^, ^9^, ^10^, ^11^

**Table 1:** Immunohistochemical expression of MUC5AC, MUC6, and HIK1083 in tubular adenoma and sessile serrated lesion.

	MUC5AC (positive, *n* (%))	MUC6 (positive, *n* (%))	HIK1083 (positive, *n* (%))
Tubular adenoma	3/23 (13)**	0/23 (0)**	0/23 (0)
Sessile serrated lesion	28/30 (93)	24/30 (80)	0/30 (0)

** *p*‐value < 0.001 between tubular adenoma and sessile serrated lesion.

### The presence of terminal βGlcNAc residues and SATB2 expression in SSLs, MVHPs, and TAs

**Figure 2:**
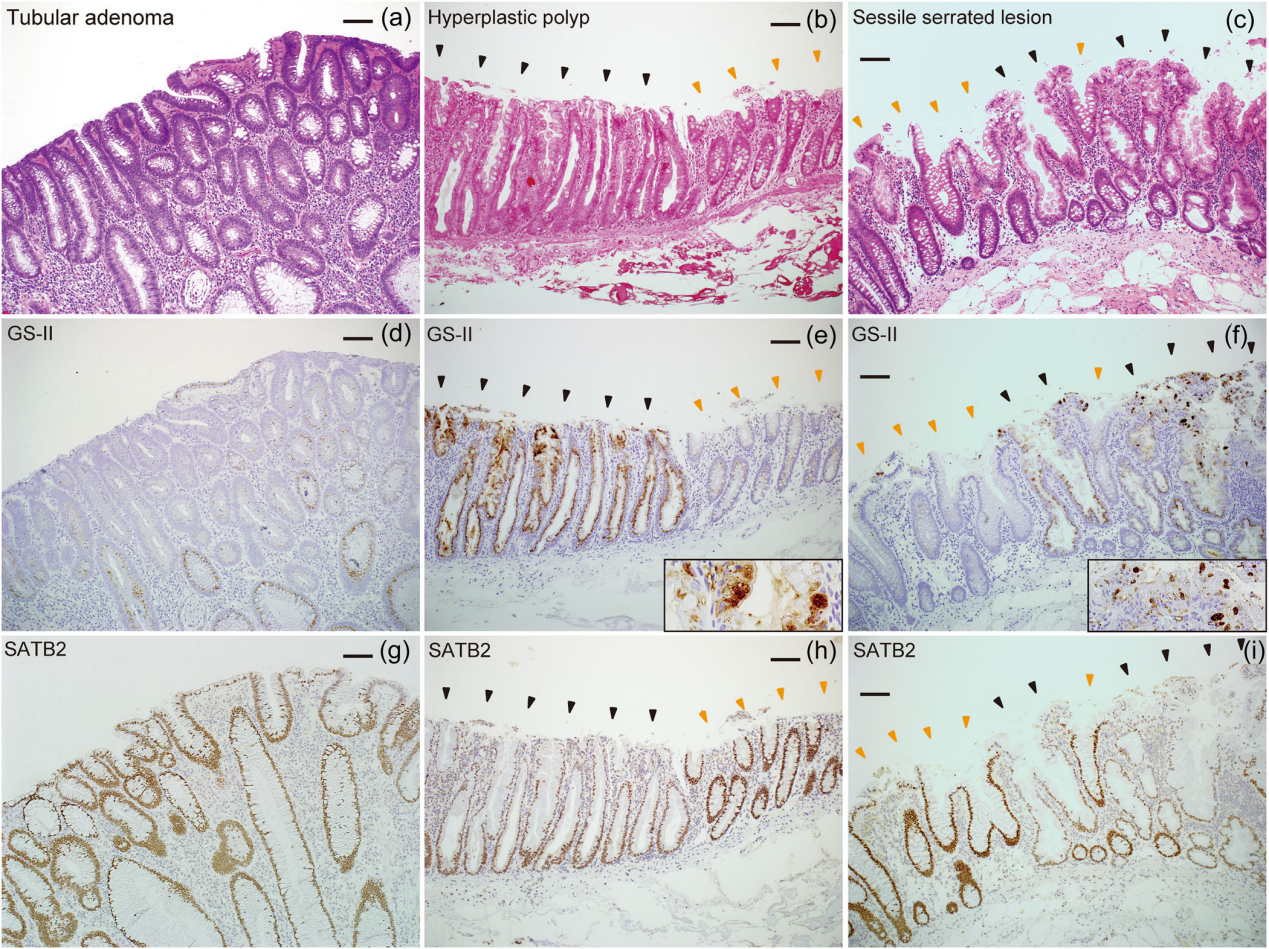
Terminal βGlcNAc residues and SATB2 expression in tubular adenomas (TAs), microvesicular hyperplastic polyps (MVHPs), and sessile serrated lesions (SSLs). (a–c) Hematoxylin and eosin staining in TAs (a), MVHPs (b), and SSLs (c). (d–f), *Griffonia simplicifolia*‐II horseradish peroxidase (GS‐II‐HRP) staining in TAs (d), MVHPs (e), and SSLs (f). (g–i) Immunostaining for SATB2 in TAs (g), MVHPs (h), and SSLs (i). Black and orange arrowheads in (b), (c), (e), (f), (h), and (i) indicate lesion sites and adjacent colonic mucosa, respectively. Scale bars, 100 μm (a–i).

We next evaluated terminal βGlcNAc residues and SATB2 expression in SSLs, MVHPs, and TAs using GS‐II‐HRP staining and SATB2 immunostaining, respectively. GS‐II‐HRP staining was almost entirely negative in normal colonic mucosa, although a small fraction of normal mucosa showed very weak positivity (orange arrowheads in Figure 2e,f). GS‐II‐HRP staining was positive in all 30 SSLs (100%) and 26 MVHPs (100%), but in only seven (30%) of 23 TAs (*p* < 0.001 between SSLs and TAs, and *p* < 0.001 between MVHPs and TAs) (Table 2, Figure 2d and black arrowheads in Figure 2e,f). Given that SSL and TA cases were HIK1083‐negative, we concluded that terminal βGlcNAc was present in these cases. GS‐II was mainly bound to the perinuclear cytoplasm, namely to the Golgi region (black arrowheads in Figure 2e,f). However, 20 (67%) of 30 GS‐II‐positive SSLs and 19 (73%) of 26 GS‐II‐positive MVHPs showed distribution to goblet cells as well, while none of the seven GS‐II‐positive TAs showed this pattern (*p* < 0.05 between SSLs and TAs, and *p* < 0.05 between MVHPs and TAs) (Table 2, Figure 2d and e,f insets). Normal colonic mucosa and all 30 SSLs, 26 MVHPs, and 23 TAs were SATB‐positive (Table 2 and Figure 2g–i). However, we observed decreased staining intensity at lesion sites relative to adjacent colonic mucosa in 21 (70%) of 30 SSLs, 12 (46%) of 26 MVHPs, and in only two (9%) of 23 TAs (*p* < 0.001 between SSLs and TAs, and *p* < 0.05 between MVHPs and TAs) (Table 2, Figure 2g and black and orange arrowheads in Figure 2h,i). Overall, relative to TAs, SSLs and MVHPs exhibited an increase in terminal βGlcNAc as well as SATB2 downregulation. In addition, SATB2 downregulation was gradually enhanced during the progression from MVHPs to SSLs, although there was no statistically significant difference between MVHPs and SSLs.

**Table 2:** GS‐II‐HRP staining and SATB2 expression in tubular adenoma, microvesicular hyperplastic polyp, and sessile serrated lesion.

	GS‐II (positive, *n* (%))	Distribution of GS‐II to goblet cells (positive, *n* (%))	SATB2 (positive, *n* (%))	SATB2 downregulation at the lesion site (positive, *n* (%))
Tubular adenoma	7/23 (30)**, ††	0/7 (0)*, †	23/23 (100)	2/23 (9)*, ††
Hyperplastic polyp	26/26 (100)	19/26 (73)	26/26 (100)	12/26 (46)
Sessile serrated lesion	30/30 (100)	20/30 (67)	30/30 (100)	21/30 (70)

Abbreviations: GS‐II, *Griffonia simplicifolia*‐II; HRP, horseradish peroxidase; SATB2, special AT‐rich sequence binding protein 2.

* *p*‐value < 0.05.

** *p*‐value < 0.001 between tubular adenoma and hyperplastic polyp.

† *p*‐value < 0.05.

†† *p*‐value < 0.001 between tubular adenoma and sessile serrated lesion.

### GS‐II‐HRP staining with N‐glycosidase digestion in SSLs, MVHPs, and TAs

**Figure 3:**
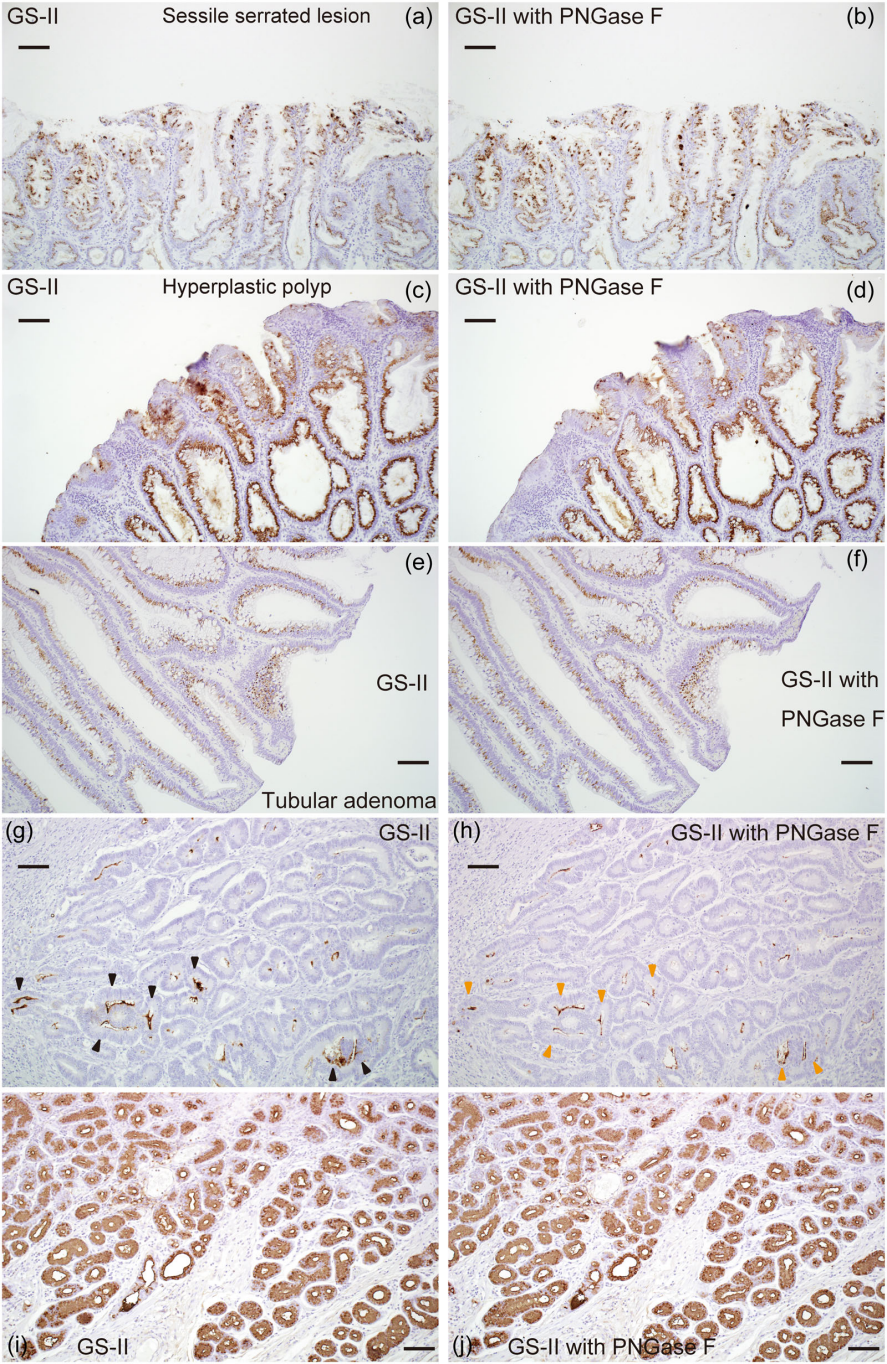
*Griffonia simplicifolia*‐II horseradish peroxidase (GS‐II‐HRP) staining of sessile serrated lesions (SSLs), microvesicular hyperplastic polyps (MVHPs), and tubular adenomas (TAs) in the presence of N‐glycosidase digestion. (a), (b) GS‐II‐HRP staining in SSLs without (a) and with (b) *N*‐glycosidase digestion. (c), (d) GS‐II‐HRP staining in MVHPs without (c) and with (d) *N*‐glycosidase digestion. (e), (f) GS‐II‐HRP staining in TAs without (e) and with (f) *N*‐glycosidase digestion. (g), (h) GS‐II‐HRP staining of colorectal carcinoma without (g) and with (h) *N*‐glycosidase digestion. Black arrowheads in (g) and orange arrowheads in (h) indicate respective positive sites. (i), (j) GS‐II‐HRP staining in gastric pyloric glands without (i) and with (j) *N*‐glycosidase digestion. Scale bars, 100 μm (a–j).

To determine whether terminal βGlcNAc found in SSLs, MVHPs, and TAs was located on *O*‐ or *N*‐glycans, we performed GS‐II‐HRP staining with PNGase F (one form of *N*‐glycosidase) digestion in these cases. GS‐II‐HRP staining in five SSLs, five MVHPs, and five TAs was virtually unchanged by PNGase F digestion (Figure 3a–f). GS‐II‐HRP staining on the luminal surface of colorectal cancer cells was partially attenuated by PNGase F digestion but did not disappear completely (black arrowheads in Figure 3g and orange arrowheads in Figure 3h). GS‐II‐HRP staining in gastric pyloric glands, which contain terminal αGlcNAc located on *O*‐glycans, was unchanged by PNGase F digestion (Figure 3i,j). These results suggest that SSLs, MVHPs, and TAs contain terminal βGlcNAc as *O*‐glycans because the staining of these cases was unchanged by PNGase F digestion even under circumstances in which the staining of colorectal cancer cells was attenuated.

## DISCUSSION

SSLs and MVHPs are precursor lesions of serrated pathway‐associated colorectal carcinomas that display gastric differentiation in mucin core protein expression.^7^, ^8^, ^9^, ^10^, ^11^ Here, we reveal that increased GS‐II binding plus SATB2 downregulation occur specifically in SSLs and MVHPs. These results confirm gastric differentiation of SSLs and MVHPs in terms of transcription factor expression and also indicate that glycosylation in SSLs is incomplete.

GS‐II lectin specifically reacts with α/βGlcNAc residues at non‐reducing ends of glycans.^12^ When we stained SSLs with HIK1083, an antibody specific for αGlcNAc, we found they were HIK1083‐negative (see Figure 1h), indicating that the GS‐II‐linked glycans observed on these lesions are βGlcNAc.

Mucin core proteins contain hundreds of heterogenous *O*‐glycans attached to the protein scaffold and may also carry a small number of *N*‐glycans (Supporting Information: supplementary Figure 1A). We previously reported that GS‐II binds to the luminal surface of colorectal cancer cells, and that reactivity of GS‐II to glycoproteins extracted from colorectal cancer tissues was almost completely abolished by PNGase F treatment, indicating that GS‐II‐responsive glycans in these tissues are located on *N*‐glycans.^13^, ^14^, ^25^ On the other hand, others report a decrease in several complex glycans and an increase in a subset of smaller glycans with terminal βGlcNAc located on *O*‐glycans in colonic mucosa of active ulcerative colitis.^26^ Considering similarities between IBD associated dysplasia, SSL, and MVHP, such as expression of gastric‐type mucin core proteins,^7^, ^8^, ^9^, ^10^, ^11^, ^27^ whether terminal βGlcNAc found in SSLs and MVHPs was located on *O*‐ or *N*‐glycans seemed to remain unclear. We found that GS‐II‐HRP staining of the luminal surface of colorectal cancer cells was partially attenuated by PNGase F digestion, although that staining did not completely disappear, unlike our previous observations seen in colorectal carcinoma tissue lysates^13^, ^14^, ^25^ (see Figure 3g,h). Such residual staining may be a technical issue related to the difficulty in completely denaturing proteins in paraffin sections, as compared with tissue lysates. However, the fact that GS‐II‐HRP staining in SSLs, MVHPs, and TAs was not attenuated under this staining condition suggests that at least part of terminal βGlcNAc in these cases is present on *O*‐glycans resistant to PNGase F digestion. Further investigation will be needed to clarify the type and amount of glycans on which βGlcNAc is located.

*O*‐linked αGalNAc attached to Ser/Thr is the initiating sugar of *O*‐glycans and is usually extended to form one of the four common core structures (Supporting Information: supplementary Figure 1B). In mammalian cells, terminal βGlcNAc residues in core two–four structures are often galactosylated to generate a Gal/GlcNAc disaccharide unit, called *N*‐acetyllactosamine (LacNAc). Each core can be extended forming poly‐LacNAc chains, which are further modified by subsequent glycosylation to form mature linear or branched *O*‐glycans^28^ (Supporting Information: supplementary Figure 1B). Therefore, terminal βGlcNAc residues are rarely found in mature *O*‐glycans. In this study, we found that terminal βGlcNAc on *O*‐glycans was increased in SSLs and MVHPs relative to TAs, and only SSLs and MVHPs showed distribution of terminal βGlcNAc to goblet cells (see Figures 2d–f and 3). This means that terminal βGlcNAc is present not only in mucin in the process of synthesis but also in the secreted mucins in SSLs and MVHPs, indicating that glycosylation in SSLs and MVHPs is incomplete. The increase in terminal βGlcNAc could be due to decreased galactosylation of βGlcNAc residues resulting from decreased galactosyltransferase activity or an increase in βGlcNAc residues themselves resulting from increased N‐acetylglucosaminyltransferase activity. Further analysis will be needed to clarify the mechanism of terminal βGlcNAc increase and the functional significance of incomplete glycosylation in SSLs and MVHPs.

It is also noteworthy that we observed a SATB2 downregulation in SSLs and MVHPs (see Figure 2 and Table 2). SATB2 downregulation has been reported in sessile serrated pathway‐associated colorectal carcinomas, IBD associated colorectal carcinomas and dysplasias,^20^, ^21^, ^22^ and here we reveal that it also occurs in SSLs and MVHPs (see Figure 2 and Table 2). SSLs and MVHPs are precursor lesions of serrated pathway‐associated colorectal carcinomas, and MVHPs are thought to gradually progress to SSLs. In this study, we revealed that SATB2 was gradually downregulated through the progression from MVHPs to SSLs, although it was not statistically significant (see Figure 2 and Table 2). Previous reports showed that Ki‐67‐positive proliferative cells become more unevenly distributed to the upper crypt portion through the progression from MVHPs to SSLs, suggesting that this increase and patchy distribution of proliferative cells may be the cause of crypt dilation, distortion, and branching in SSLs.^29^, ^30^, ^31^ Therefore, the gradual SATB2 downregulation through the progression from MVHPs to SSLs may contribute to the morphological changes described above by leading to a gradual deterioration of intestinal differentiation and disruption of the distribution of the proliferative zone. Further studies will be required to determine the relationship between SATB2 downregulation, gastric differentiation, and changes in the proliferative zone and morphology in SSLs.

Regarding the relationship between increased terminal βGlcNAc and SATB2 downregulation, the former occurred early in MVHPs and almost unchanged in SSLs, while the latter was gradually enhanced during the progression from MVHPs to SSLs. Therefore, the mechanisms involved in terminal βGlcNAc increase and SATB2 downregulation may differ. CDX2 expression is reportedly retained in SSLs but later decreases in serrated pathway‐associated carcinomas;^11^, ^32^ thus mechanisms downregulating SATB2 and CDX2 may also differ. In serrated pathway‐associated colorectal carcinogenesis, lesions bearing activating *BRAF* mutations develop into MVHPs, further develop into SSLs as promoter regions of several genes become methylated, and finally develop into MSI‐H and CIMP‐H colorectal carcinomas once hMLH1 is inactivated by methylation of the promoter region.^6^ Therefore, terminal βGlcNAc increase early in MVHPs may be due to *BRAF* mutation. On the other hand, SATB2 downregulation in MVHPs and SSLs may be due to methylation of the promoter region that occurs gradually during the progression from MVHPs to SSLs. CDX2 downregulation in MSI‐H and CIMP‐H colorectal carcinomas may also be due to methylation of the promoter region, but it may occur later than SATB2 for unknown reasons. Further investigation will be required to determine precise mechanisms underlying terminal βGlcNAc increase, SATB2 downregulation, and CDX2 downregulation.

In conclusion, we have newly identified increased terminal βGlcNAc combined with SATB2 downregulation as biological features of SSLs and MVHPs. These features can serve as SSL and MVHP biomarkers and have implications for serrated pathway tumorigenesis.

## AUTHOR CONTRIBUTIONS

Hisanori Matoba designed the research, performed immunostaining, analyzed histopathological data, constructed figures and tables, and drafted the manuscript. Mai Iwaya provided suggestions for experimental design and analyzed histopathological data. Yoshiko Sato performed immunostaining. Jun Nakayama provided suggestions for the experimental design and manuscript drafting. Noriyasu Kobayashi, Haruka Takemura, Yusuke Kouno, and Ayumi Karasawa helped prepare the cases.

## CONFLICT OF INTEREST STATEMENT

The authors declare no conflict of interest.

## Supporting information

Supporting information.

Supporting information.

## References

[1] Weisenberger DJ , Siegmund KD , Campan M , Young J , Long TI , Faasse MA , et al. CpG island methylator phenotype underlies sporadic microsatellite instability and is tightly associated with *BRAF* mutation in colorectal cancer. Nature Genet. 2006;38:787–93. 16804544 10.1038/ng1834

[2] Kambara T , Simms LA , Whitehall VLJ , Spring KJ , Wynter CVA , Walsh MD , et al. *BRAF* mutation is associated with DNA methylation in serrated polyps and cancers of the colorectum. Gut. 2004;53:1137–44. 15247181 10.1136/gut.2003.037671 PMC1774130

[3] Nosho K , Irahara N , Shima K , Kure S , Kirkner GJ , Schernhammer ES , et al. Comprehensive biostatistical analysis of CpG island methylator phenotype in colorectal cancer using a large population‐based sample. PLoS One. 2008;3:e3698. 10.1371/journal.pone.0003698 19002263 PMC2579485

[4] Qi Li W , Kawakami K , Ruszkiewicz A , Bennett G , Moore J , Iacopetta B . *BRAF* mutations are associated with distinctive clinical, pathological and molecular features of colorectal cancer independently of microsatellite instability status. Mol Cancer. 2006;5:2. 10.1186/1476-4598-5-2 16403224 PMC1360090

[5] Samowitz WS , Albertsen H , Herrick J , Levin TR , Sweeney C , Murtaugh MA , et al. Evaluation of a large, population‐based sample supports a CpG island methylator phenotype in colon cancer. Gastroenterology. 2005;129:837–45. 16143123 10.1053/j.gastro.2005.06.020

[6] Snover DC . Update on the serrated pathway to colorectal carcinoma. Hum Pathol. 2011;42:1–10. 20869746 10.1016/j.humpath.2010.06.002

[7] Hirono H , Ajioka Y , Watanabe H , Baba Y , Tozawa E , Nishikura K , et al. Bidirectional gastric differentiation in cellular mucin phenotype (foveolar and pyloric) in serrated adenoma and hyperplastic polyp of the colorectum. Pathol Int. 2004;54:401–7. 15144398 10.1111/j.1440-1827.2004.01639.x

[8] Mochizuka A , Uehara T , Nakamura T , Kobayashi Y , Ota H . Hyperplastic polyps and sessile serrated “adenomas” of the colon and rectum display gastric pyloric differentiation. Histochem Cell Biol. 2007;128:445–55. 17851679 10.1007/s00418-007-0326-2

[9] Gibson JA , Hahn HP , Shahsafaei A , Odze RD . MUC expression in hyperplastic and serrated colonic polyps: lack of specificity of MUC6. Am J Surg Pathol. 2011;35:742–9. 21490447 10.1097/PAS.0b013e31821537a2

[10] Fujita K , Hirahashi M , Yamamoto H , Matsumoto T , Gushima M , Oda Y , et al. Mucin core protein expression in serrated polyps of the large intestine. Virchows Arch. 2010;457:443–9. 20803031 10.1007/s00428-010-0959-8

[11] Walsh MD , Clendenning M , Williamson E , Pearson SA , Walters RJ , Nagler B , et al. Expression of MUC2, MUC5AC, MUC5B, and MUC6 mucins in colorectal cancers and their association with the CpG island methylator phenotype. Mod Pathol. 2013;26:1642–56. 23807779 10.1038/modpathol.2013.101

[12] Iyer PNS , Wilkinson KD , Goldstein IJ . An N‐acetyl‐D‐glucosamine binding lectin from Bandeiraea Simplicifolia seeds. Arch Biochem Biophys. 1976;177:330–33. 999292 10.1016/0003-9861(76)90444-6

[13] Nakayama J , Katsuyama T , Ono K , Honda T , Akamatsu T , Hattori H . Large bowel carcinoma‐specific antigens detected by the lectin, Griffonia simplicifolia agglutinin‐II. Jpn J Cancer Res (Gann). 1985;76:1078–84. 2418001

[14] Ota H , Nakayama J , Katsuyama T , Kanai M . Histochemical comparison of specificity of three bowel carcinoma‐reactive lectins, Griffonia simplicifolia agglutinin‐II, peanut agglutinin and Ulex europaeus agglutinin‐I. Acta Pathol Jpn. 1988;38:1547–59. 3239392 10.1111/j.1440-1827.1988.tb02294.x

[15] Dobreva G , Dambacher J , Grosschedl R . SUMO modification of a novel MAR‐binding protein, SATB2, modulates immunoglobulin μ gene expression. Genes Dev. 2003;17:3048–61. 14701874 10.1101/gad.1153003 PMC305257

[16] FitzPatrick DR , Carr IM , McLaren L , Leek JP , Wightman P , Williamson K , et al. Identification of SATB2 as the cleft palate gene on 2q32‐q33. Hum Mol Gen. 2003;12:2491–501. 12915443 10.1093/hmg/ddg248

[17] Lin F , Shi J , Zhu S , Chen Z , Li A , Chen T , et al. Cadherin‐17 and SATB2 are sensitive and specific immunomarkers for medullary carcinoma of the large intestine. Arch Pathol Lab Med. 2014;138:1015–26. 24437456 10.5858/arpa.2013-0452-OA

[18] Magnusson K , De Wit M , Brennan DJ , Johnson LB , McGee SF , Lundberg E , et al. SATB2 in combination with cytokeratin 20 identifies over 95% of all colorectal carcinomas. Am J Surg Pathol. 2011;35:937–48. 21677534 10.1097/PAS.0b013e31821c3dae

[19] Moh M , Krings G , Ates D , Aysal A , Kim GE , Rabban JT . SATB2 expression distinguishes ovarian metastases of colorectal and appendiceal origin from primary ovarian tumors of mucinous or endometrioid type. Am J Surg Pathol. 2016;40:419–32. 26551622 10.1097/PAS.0000000000000553

[20] Ma C , Olevian DC , Lowenthal BM , Jayachandran P , Kozak MM , Chang DT , et al. Loss of SATB2 expression in colorectal carcinoma is associated with DNA mismatch repair protein deficiency and *BRAF* mutation. Am J Surg Pathol. 2018;42:1409–17. 30001238 10.1097/PAS.0000000000001116

[21] Iwaya M , Ota H , Tateishi Y , Nakajima T , Riddell R , Conner JR . Colitis‐associated colorectal adenocarcinomas are frequently associated with non‐intestinal mucin profiles and loss of SATB2 expression. Mod Pathol. 2019;32:884–92. 30710095 10.1038/s41379-018-0198-0

[22] Ma C , Henn P , Miller C , Herbst C , Hartman DJ , Pai RK . Loss of SATB2 expression is a biomarker of inflammatory bowel disease‐associated colorectal dysplasia and adenocarcinoma. Am J Surg Pathol. 2019;43:1314–22. 31318711 10.1097/PAS.0000000000001330

[23] WHO Classification of Tumours Editorial Board the WHO Classification of Tumours . Digestive system tumours. Volume 1. 5th ed. Geneva: World Health Organization; 2019. p. 163–73.

[24] Kanda Y . Investigation of the freely available easy‐to‐use software “EZR” for medical statistics. Bone Marrow Transplant. 2013;48:452–58. 23208313 10.1038/bmt.2012.244 PMC3590441

[25] Nakayama J , Okano A , Maeda H , Miyachi M , Ota H , Katsuyama T , et al. Griffonia simplicifolia agglutinin‐2‐binding glycoprotein as a novel carbohydrate antigen of human colonic carcinoma. Jpn J Cancer Res. 1990;81:388–95. 1694841 10.1111/j.1349-7006.1990.tb02580.x PMC5918055

[26] Larsson JMH , Karlsson H , Crespo JG , Johansson MEV , Eklund L , Sjövall H , et al. Altered O‐glycosylation profile of MUC2 mucin occurs in active ulcerative colitis and is associated with increased inflammation. Inflamm Bowel Dis. 2011;17:2299–307. 21290483 10.1002/ibd.21625

[27] Tatsumi N , Kushima R , Vieth M , Mukaisho K , Kakinoki R , Okabe H , et al. Cytokeratin 7/20 and mucin core protein expression in ulcerative colitis‐associated colorectal neoplasms. Virchows Arch. 2006;448:756–62. 16609910 10.1007/s00428-006-0188-3

[28] Varki A , Cummings RD , Esko JD , Stanley P , Hart GW . Essentials of glycobiology. 3rd ed. New York: Cold Spring Harbor Laboratory Press; 2017. p. 99–123. 20301239

[29] Hisamatsu K , Noguchi K , Tomita H , Muto A , Yamada N , Kobayashi K , et al. Distinctive crypt shape in a sessile serrated adenoma/polyp: distribution of Ki67‐, p16INK4a‐, WNT5A‐positive cells and intraepithelial lymphocytes. Oncol Rep. 2017;38:775–84. 28627675 10.3892/or.2017.5725 PMC5561931

[30] Fortuna D , Boman B , O'Neill R , Palazzo J . MCM2 expression in serrated polyps demonstrates aberrant cellular proliferation. Hum Pathol. 2017;63:177–83. 28302537 10.1016/j.humpath.2017.02.020

[31] Torlakovic EE , Gomez JD , Driman DK , Parfitt JR , Wang C , Benerjee T , et al. Sessile serrated adenoma (SSA) vs. traditional serrated adenoma (TSA). Am J Surg Pathol. 2008;32:21–9. 18162766 10.1097/PAS.0b013e318157f002

[32] Kim JH , Kim KJ , Rhee YY , Bae JM , Cho NY , Lee HS , et al. Gastric‐type expression signature in serrated pathway‐associated colorectal tumors. Hum Pathol. 2015;46:643–56. 25704805 10.1016/j.humpath.2015.01.003

