# Supplementary material for: Increased GS‐II lectin binding and SATB2 downregulation are biological features for sessile serrated lesions and microvesicular hyperplastic polyps
Source: Pathol Int. 2023 Apr 10;73(6):246–54. doi: 10.1111/pin.13321 (PMC11551811; doi:10.1111/pin.13321)
Supplement: Supplementary file 1 — Supporting information. [file PIN-73-246-s002.docx]

**
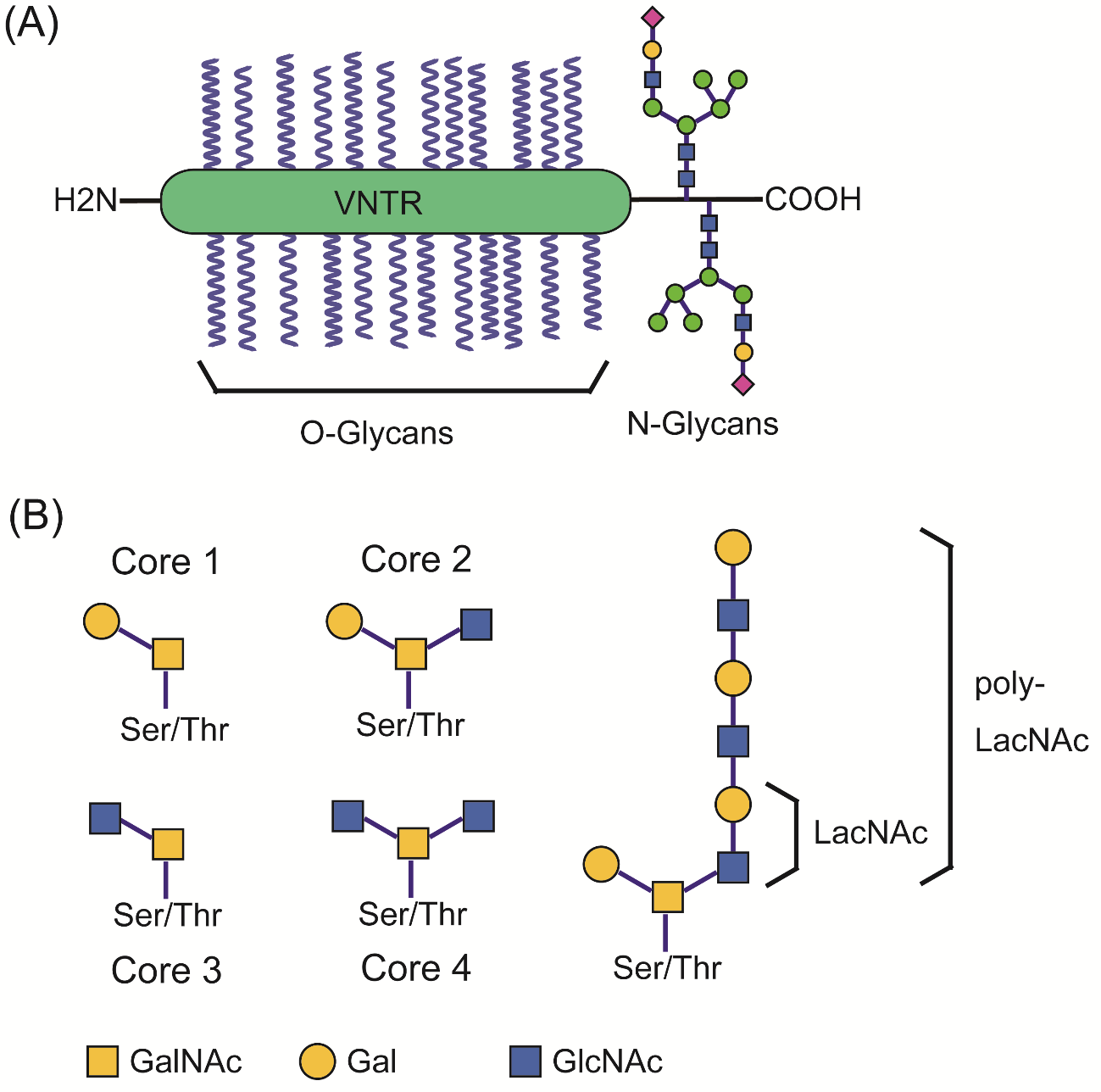
**

**Supplementary Figure 1**

**(A)** A simplified model of secreted mucin. The variable number tandem repeat (VNTR) region rich in Ser, Thr, and Pro residues is highly *O*-glycosylated, as represented by blue zigzag lines. A smaller number of *N*-glycans is present at the carboxyl terminus. **(B)** Core 1-4 structure in *O*-glycans and its extension by poly-*N*-acetyllactosamine (LacNAc) formation. Yellow-filled squares indicate GalNAc, yellow-filled circles indicate Gal, and blue-filled squares indicate GlcNAc.
